# Prevalence of Needle Stick Injuries Among Healthcare Workers at a Tertiary Care Centre in Kochi, India

**DOI:** 10.7759/cureus.72077

**Published:** 2024-10-21

**Authors:** Anand Raj, Ali Haider, Agraja J, Anjali A, Andrew A, Aparna S, Navami Sasidharan, Melvi Johnson, Aswathy S

**Affiliations:** 1 Community Medicine, Amrita Institute of Medical Sciences and Research Centre, Kochi, IND

**Keywords:** healthcare workers, needlestick injury, post exposure prophylaxis, prevalence, recapping of needles

## Abstract

Background

Needle stick injuries (NSIs) are a prevalent occupational hazard among healthcare workers (HCWs) globally. Strict adherence to standard work precautions by all HCWs at all times and the implementation of post-exposure prophylaxis (PEP) are measures for reducing the occurrence of NSIs. However, NSIs are poorly documented in the literature and may be a cause of morbidity in the long run.

Objectives

Our study aims to determine the prevalence of NSIs among HCWs and its associated factors in a tertiary healthcare centre in Kerala, India.

Methods

A cross-sectional study was conducted among 210 consenting HCWs who worked in different blocks of a tertiary care hospital in the Ernakulam district, Kerala, India. Data was collected using a semi-structured questionnaire through personal interviews. The questionnaire gathered sociodemographic information, details regarding NSI occurrences over the past one year, the devices involved and PEP. Data analysis was performed using Statistical Package for the Social Sciences (IBM SPSS Statistics for Windows, IBM Corp., Version 20.0, Armonk, NY), and categorical variables were expressed as frequencies and percentages. The chi-square test and binomial logistic regression were employed to identify factors associated with NSIs.

Results

The prevalence of NSIs among HCWs in the past 12 months was 31% (65 out of 210), with an average of 1.34 NSIs per HCW per year. The most commonly involved device was the intravenous (IV) cannula, and 22 out of 65 (33.8%) injuries occurred during injection procedures. A majority (45 out of 65; 69.23%) of HCWs reported NSI and 51 of them (78.4%) received PEP. Logistic regression analysis showed that HCWs aged over 31 years (adjusted odds ratio (AOR): 3.39; 95% confidence interval (CI): 1.29-8.42) were independently associated with a higher occurrence of NSIs.

Conclusion

Our findings underscore the importance of implementing comprehensive safety measures, including strict adherence to standard work precautions and timely administration of PEP.

## Introduction

Needle stick injuries (NSIs) are a significant occupational hazard faced by healthcare workers (HCWs) worldwide [1,2]. An NSI refers to the piercing of the skin by a needle or any sharp object that had prior contact with another individual’s blood, tissue, or body fluids before the actual injury occurred [3]. These incidents are commonly reported in healthcare settings, affecting a substantial number of HCWs. According to the World Health Organization (WHO), over two million of the total 35 million HCWs worldwide experience occupational exposure to NSIs each year [4]. Such injuries often occur during procedures like needle recapping and blood sample collection [5]. NSIs pose a risk of transmitting blood-borne pathogens, including human immunodeficiency virus (HIV), hepatitis B virus (HBV) and hepatitis C virus (HCV), which are of significant concern due to their severe complications. The risk of contracting these diseases following percutaneous exposure among HCWs is significant, with transmission rates of 37%, 39%, and 4.4% for HBV, HCV, and HIV, respectively [6].

In India, the prevalence of NSIs among HCWs is alarmingly high, with studies reporting rates as high as 58% [7] and 73% [8]. Factors contributing to this include inadequate training, poor adherence to safety protocols, and unsafe practices such as needle recapping [5,9,10]. Most of these injuries can be prevented by minimizing the unnecessary use of needles, implementing devices equipped with safety features, and promoting education and safe handling practices [11]. Many times, these injuries are not reported. The failure to report NSIs promptly leads to delays in receiving post-exposure prophylaxis (PEP), which in turn increases the risk of infection. PEP, which must be initiated within 72 hours of exposure, is crucial for preventing infection and includes follow-up testing at three and six months post-exposure [12].

Despite the high prevalence of NSIs among HCWs, there is limited literature on this topic in the context of Kerala, India. Given that Ernakulam has numerous tertiary care hospitals, conducting this study would help to bridge the existing gaps concerning NSIs. Thus, this study aims to determine the prevalence of NSIs among HCWs and its associated factors in a tertiary care hospital setting.

## Materials and methods

A cross-sectional study was conducted to determine the prevalence of NSIs among HCWs in a tertiary care hospital in Kerala from February 2023 to July 2023. The inclusion criteria were the male and female HCWs from those departments who routinely handle needles and sharps. The HCWs from non-clinical departments where needle use is uncommon were excluded from the study. The study population consisted of doctors, nurses, biomedical waste collectors and lab technicians working across different blocks of the hospital. Each block, namely A, B, C, and D was treated as a cluster.

The sample size calculation was based on a study done by Sharma et al., which reported an NSI prevalence of 79.5% among HCWs in Delhi [13]. Using the formula $n = \frac{4pq}{d^2}$ with a 6% absolute error, the required sample size was calculated to be 182. Assuming a non-response rate of 10%, the final sample size was adjusted to 202. Finally, data was obtained from 210 consenting HCWs.

The study was conducted across four blocks of the hospital, namely A, B, C, and D, with each block treated as a cluster. Data collection in each block took place over three days, between 10 a.m. and 12 p.m., which coincided with the hospital's busiest hours. In blocks A, B, and D, a total of 65, 57, and 69 HCWs were approached, with 53, 52, and 54, respectively, consenting to participate. In block C, 63 HCWs were approached, with 51 agreeing to participate.

A self-designed, semi-structured questionnaire was used to assess the prevalence of NSI among HCWs and the factors associated with it. It included demographic details, HCW’s work category, information regarding the type of injury, the source of injury, the procedure during which the injury occurred, the device involved, details of their post-exposure measures taken, and whether they underwent testing for HBV, HCV and HIV.

Data was entered into MS Excel (Microsoft® Corp., Redmond, WA, USA) and analyzed using Statistical Package for the Social Sciences (IBM SPSS Statistics for Windows, IBM Corp., Version 20.0, Armonk, NY). Categorical variables were expressed as frequencies and percentages with a 95% confidence interval (CI). A chi-square test was used to find the factors associated with NSI. Binomial logistical regression was used to determine the independent variables associated with NSIs. A p-value less than 0.05 was considered to be significant.

## Results

A total of 210 HCWs from different blocks of the hospital took part in the study. The mean age of the participants was 31 ± 6 years, with an age range from 23 to 52 years. Among the participants more than half (110 out of 210; 52.4%) were under the age of 31 and 143 out of 210 (68.1%) participants were females. More than a third (75 out of 210; 35%) of them were nurses and the mean working experience of participants was 6 ± 5 years (Table 1).

**Table 1:** Sociodemographic characteristics of the study population (n=210)

Category	Frequency (n)	Percentage (%)
Age in years		
Less than or equal to 31 years	110	52.4
Above 31 years	100	47.6
Gender		
Males	67	31.9
Females	143	68.1
Healthcare worker category		
Biomedical waste collectors	10	4.8
Lab technicians	39	18.6
Nurse	75	35.7
Interns	17	8.1
Doctors	69	32.9
Experience in years		
Less than or equal to six years	128	61
More than six years	82	39

About 65 (31%) HCWs had experienced at least an event of NSI in the last 12 months. Among those individuals who experienced an NSI, only a single event was reported by 46 (70.7%). The average NSI per HCW per year was 1.34. Among the reported NSI incidents, the majority of injuries occurred among nurses, with 26 events per year, followed by doctors, who reported 17 events per year (Table 2).

**Table 2:** Number of events per HCW category in the last year HCW: healthcare worker; NSI: needle stick injury

HCW category	Number of NSI events/year, presented as n (%)		
	1	2	3
Biomedical waste collectors and lab technician	11 (22.4)	4 (8.2)	1 (2.0)
Nurses	22 (29.3)	4 (5.3)	0
Interns	4 (23.5)	1 (5.9)	1 (5.9)
Doctors	9 (13)	7 (10.1)	1 (1.4)

The majority of the participants reported that injection (22 out of 65; 33.8%) caused NSI followed by recapping needles (17 out of 65; 26.1%). The majority of HCWs (27 out of 65; 31%) attributed the cause of NSI to various reasons such as inattention followed by heavy workload (16 out of 65; 24.6%). About 44 (67.7%) HCWs sought medical attention as an immediate response following an event of NSI and 51 (78.6%) of them received PEP. About 20 out of 65 (30%) HCWs did not get tested for HBV, HCV and HIV. Only eight (12.37%) underwent testing at the time of exposure, three months and six months after the exposure (Table 3).

**Table 3:** Needle stick injury incurred by healthcare workers (n=65) NSI: needle stick injury; HBV: hepatitis B virus; HCV: hepatitis C virus; HIV: human immunodeficiency virus

Needle stick injury	Frequency (n=65)	Percentage (%)
Site of injury		
Finger	55	84.6
Hand/palm	6	9.23
Forearm/others	4	6.15
Severity of injury		
Superficial (little/no bleeding)	25	38.46
Moderate (skin punctured with little bleeding)	37	56.92
Severe (deep prick/cut with profuse bleeding)	3	4.615
Device involved		
Hypodermic needle	16	24.61
Intravenous (IV) cannula	23	35.38
Lancet/racers/scissors	14	21.54
Suture needles	8	12.54
Butterfly needle	4	6.15
Procedure during which the NSI occurred		
During injection procedures	22	33.8
Recapping needle	17	26.1
During IV Line insertion	5	7.7
During disposal to sharps container	14	21.54
During surgical procedure	7	10.76
Perceived cause of NSI		
Hasty work and inattention	27	41.53
Tiredness	8	12.31
Lack of personal protective equipment	14	21.54
Heavy workload	16	24.6
Immediate response to NSI		
Did nothing	3	4.62
Licked the wound site	2	3.07
Washed with soap and water	16	24.61
Sought medical attention	44	67.7
Reported NSI		
Yes	45	69.23
No	20	30.77
Received post-exposure prophylaxis		
Yes	51	78.46
No	14	21.53
Testing status		
Not tested	20	30.77
Did testing for HBV, HCV, HIV at the time of exposure	34	52.30
Did testing for HBV, HCV, HIV at the time of exposure and after three months	3	4.61
Did testing for HBV, HCV, HIV at the time, at three and six months of exposure	8	12.31

In the bivariate analysis, the HCWs in the 31 year and above category had a significantly higher proportion of NSI. There was also a significantly higher proportion of NSI among HCWs having experience of more than six years. Multivariable regression analysis showed that age above 31 years had a three times higher probability (adjusted odds ratio (AOR): 3.39; 95% CI: 1.29-8.42) of experiencing an NSI (Table 4).

**Table 4:** Independent determinants of needlestick injury among healthcare workers * p < 0.05 indicates a significant association; # reference category

Variable	Yes	No	Odds ratio	p-value	Adjusted OR (confidence interval)	p-value
Age in years						
Less than or equal to 31 years^#^	25 (22.7)	85 (77.3)	2.26	0.007	1	0.013^*^
Above 31 years	40 (40)	60 (60)			3.07 (1.28-6.42)	
Gender						
Male^#^	25 (37.3)	42 (62.7)	0.65	0.172	-	-
Female	40 (62.7)	103 (72)				
Experience (in years)						
Less than or equal to six years^#^	33 (25.8)	95 (74.2)	1.84	0.043	1	0.843
Above six years	32 (39)	50 (61.0)			1.97 (0.45-4.62)	
Healthcare worker category						
Lab technicians and Biomedical waste collectors	16 (32.7)	33 (67.3)	-	0.575	0.69 (0.85-0.38)	0.69
Nurse	26 (34.7)	49 (65.3)			0.54 (0.16-1.85)	0.33
Interns	6 (35.3)	11 (64.7)			1.97 (0.83-4.62)	0.12
Doctors^#^	17 (24.6)	52 (75.4)			1	

## Discussion

In the present study, the proportion of NSIs among HCWs was found to be 31%, with an average of 1.34 NSIs per HCW per year. We also observed that age (above 31 years) was significantly associated with the occurrence of NSI. Studies from various parts of India have shown that the proportion of NSI ranges from 52.6% [13] to 79.5% [12]. Diverse international studies also have demonstrated varying rates of NSI. A study conducted in Pakistan [14] indicated an NSI prevalence of 70%, whereas another study from Malaysia reported a prevalence of 52.9% [15] among HCWs. The prevalence of NSI in our study is lower as compared to the studies done both nationally and internationally. This disparity could be due to differences in the working environment and standard operating protocols. Our study found the average number of NSIs per HCW in the past year to be 1.34, which is lower than the 3.85 average reported in a tertiary hospital in Delhi [12]. Among the HCW category, nurses had the highest number of NSIs, which is in line with another study done in North India [16].

In our study, the most common devices involved in NSIs were intravenous (IV) cannulas, followed by hypodermic needles. Several other studies showed the device involved with NSI to be hypodermic needles followed by IV cannulas [17]. We found that the most common procedure that caused NSI was during the injection procedure, which was also seen in a study done by Jameela et al. [18]. Another study based in Oman [19] identified the most likely process involved in injury to be recapping the needles (59%), which was found to be the second most common procedure in our study.

Another important finding in our study was that 69% of the HCWs who experienced NSIs reported the event, which is higher as compared to 31% reporting in Pakistan [20] and 32% in Goa [21]. This higher reporting rate of NSIs could be attributed to the strict adherence to guidelines following NSI exposure and better overall awareness and training regarding the importance of reporting such incidents [22]. In the present study, a high proportion of people (78.4%) received PEP following the injury, when compared to studies done in Karnataka (67%) [23] and Gujarat (35.9%) [16]. The majority of the HCWs (41%) reported the perceived cause of injury to be hasty work and inattention, followed by heavy workload. This may be due to the high patient volume in the tertiary care hospital where the study was conducted.

HCWs above 31 years of age were three times more likely to report NSIs in our study. This contrasts with findings from Saudi Arabia, which reported a higher incidence of NSIs among those aged 21-30 years with a decrease in incidence as age increased [24]. Conversely, another study conducted in Iran [25] also noted a high prevalence of NSIs among individuals with advancing age. These varying results suggest that the relationship between age and NSIs may vary across different regions and healthcare contexts [26].

The limitations of the study are that it lacks equal representation across various healthcare categories. Additionally, the extent of completion of the PEP course and adherence to PEP protocols among individuals could not be examined.

## Conclusions

The study revealed that HCWs over the age of 31 were significantly more likely to sustain NSIs, aligning with findings from similar studies in other regions. This highlights the need for targeted interventions, particularly focusing on older HCWs who may be at higher risk. The results emphasize the importance of improving safety practices during procedures involving IV cannulas and hypodermic needles, which were most commonly associated with NSIs. Further, larger studies are necessary to identify gaps in current safety protocols. Strengthening training programs, addressing workload concerns, and improving adherence to standard operating protocols are essential steps toward reducing the incidence of NSIs among HCWs.

## Appendices

**Table 5:** Questionnaire

Questionnaire		
1	Age in years	………………………
2	Gender	Male
		Female
		Others
3	Type of healthcare worker	Doctor
		Nurse
		Lab technician
		Biomedical waste collectors
		Others………
4	Years of experience in current position	……………..
5	Have you had any needle stick injury in past one year?	Yes
		No
6	If yes, how many needle stick injuries have you had in past one year?	………..
7	Where did the injury occur on your body?	…………….
8	What was the type of Injury in the previous NSI?	Superficial (little or no bleeding
		Moderate (skin punctured, little bleeding)
		Severe (deep stick/cut, or profuse bleeding)
9	What was the procedure during which last needle stick injury occurred?	Recapping a needle
		Surgical blade injury
		During disposing to sharps container
		During blood collection
		During IV line insertion
		During injection procedures
		If others, specify:…………………………………
10	What was the device involved in the previous injury?	Lancets/razors/scissors
		Suture needles
		Intravenous (IV) cannula:
		Butterfly needle
		Hypodermic needle
		Others …………………………….
11	What do you believe was the reason for the Injury?	Heavy workload
		Lack of protection measures
		Inattention
		Hasty work tiredness
		Others………………………
12	What was your immediate response after needle stick injury?	Washed with soap and water
		Medical attention (first aid)
		Lick the injured site
		Nothing
13	Was post-exposure prophylaxis (PEP) taken?	Yes
		No
14	If yes, what was the PEP recieved?	Took PEP treatment against HIV-AIDS
		Took PEP treatment against Hepatitis B
		Got tested for HBV/HCV/HIV at the time of exposure
		Got tested for HBV/HCV/HIV after 3 months of exposure
		Got tested for HBV/HCV/HIV after 6 months of exposure
15	Was the needle stick injury reported?	Yes
		no
